# The Roles of Growth Language Mindset, Metacognitive Strategies, and Language Learning Self-Efficacy in Predicting L2 Willingness to Communicate: A Network Analysis and a Chain Mediation Model

**DOI:** 10.3390/bs15040521

**Published:** 2025-04-13

**Authors:** Yuk-Hong Ho, Aitao Lu, Siyi Liu, Wanyi Chen, Jingtao Huang, Minyun Zheng

**Affiliations:** 1 Philosophy and Social Science Laboratory of Reading and Development in Children and Adolescents, South China Normal University, Ministry of Education, Guangzhou 510631, China; yukhongho@m.scnu.edu.cn (Y.-H.H.); 2019010225@m.scnu.edu.cn (S.L.); psychenwy@m.scnu.edu.cn (W.C.); 2022023867@m.scnu.edu.cn (J.H.); 2022023893@m.scnu.edu.cn (M.Z.); 2 School of Psychology, South China Normal University, Guangzhou 510631, China; 3 Center for Studies of Psychological Application, South China Normal University, Guangzhou 510631, China

**Keywords:** growth language mindset, metacognitive strategies, language learning self-efficacy, L2 willingness to communicate, network analysis, chain mediation model

## Abstract

While past research has affirmed the supportive role of growth language mindset (GLM) in fostering L2 willingness to communicate (L2 WTC), there remains a scarcity of studies exploring how growth language mindset may predict L2 WTC. This study aimed to investigate the impact of growth language mindset on L2 WTC while considering the chain mediating influences of metacognitive strategies and language learning self-efficacy (LLSE). Additionally, network analysis was used to examine the internal structure and connections between growth language mindset and L2 WTC. A total of 532 L2 learners (32.60% male; 67.40% female), aged between 17 and 25 years (*M* = 20.42 years; *SD* = 1.49 years), participated in an online survey to assess the variables of interest. The results revealed that growth language mindset has a positive predictive effect on L2 WTC, with specific items—GLM4 (“You can always change your L2 ability.”), GLM1 (“No matter who you are, you can significantly change your language intelligence level.”), and L2 WTC5 (“When you have an opportunity to explain in English your own culture to your classmates.”)—emerging as key indicators. The relationship between GLM and L2 WTC was sequentially mediated by metacognitive strategies and LLSE. This chain mediation model explained 85% of the variance in L2 WTC, with LLSE (*β* = 0.61) demonstrating the largest practical impact. These findings suggest that fostering metacognitive strategies and enhancing LLSE can significantly amplify the impact of a GLM on L2 WTC, highlighting meaningful effects beyond mere statistical significance.

## 1. Introduction

In today’s globalized world, second language (L2) proficiency is indispensable for navigating interconnected professional, educational, and social landscapes. Beyond mere cross-cultural communication, L2 competence enables participation in multinational collaborations (e.g., international business negotiations, academic research teams), access to global digital platforms, and engagement with diverse communities in migration-driven societies. Central to this competence is willingness to communicate (WTC) in an L2, which reflects a learner’s motivation to actively use the language despite challenges and is a critical predictor of successful acquisition (MacIntyre et al., 1998). Researchers have identified key factors shaping L2 WTC, particularly through the lens of positive psychology—a framework emphasizing strengths such as resilience, self-efficacy, and adaptive learner beliefs. For instance, studies demonstrate that psychological well-being and resilience (H. Wang et al., 2024) foster sustained engagement in L2 interactions, while positive beliefs about one’s learning capacity (e.g., growth mindset; Peng & Woodrow, 2010) enhance communicative confidence. These factors transcend statistical significance, directly informing pedagogical strategies to nurture learners’ long-term proficiency and real-world communicative agility.

Despite the acknowledgment of these positive psychological factors, there is a relative scarcity of research on how learners’ beliefs about learning specifically affect L2 WTC, particularly among Chinese L2 learners striving to master English. The significance of examining these learners’ experiences and obstacles is accentuated by insights from the Test of Oral Proficiency in English (TOPE) in mainland China, which emphasizes the shift from a focus solely on written proficiency to improving students’ oral English speaking abilities (Liu & Jia, 2017). The escalating attention on Chinese learners of English in L2 contexts has underscored the growing importance of enhancing L2 learners’ overall communicative competence (Wei & Xu, 2022). Regrettably, the limited opportunities for Chinese learners to practice their English speaking skills both within and outside classrooms exacerbate this challenge. This scenario highlights the imperative for EFL teachers to ramp up efforts in fostering practical learning experiences and for further research to grasp and support the unique hurdles confronted by these learners.

Previous research has indicated that learners’ beliefs about learning are critical to L2 WTC (Peng & Woodrow, 2010; H. Wang et al., 2024). One such study by H. Wang et al. (2021) investigated the effect of language mindset on L2 WTC in class and found that the effects of language mindset were fully mediated by enjoyment, pride, and boredom. Another study by Zarrinabadi et al. (2021) showed that growth language mindsets mediated the link between autonomy support, communicative competence, and WTC. While the impact of L2 beliefs on L2 WTC has been examined, the specific underlying mechanisms of such effects remain unclear. To bridge this gap, it is essential to consider the broader spectrum of strategies and self-beliefs that influence language learning behaviors. Research has investigated the influence of social and affective strategies on L2 WTC (e.g., Interaction: Peng, 2020; Confidence: MacIntyre & Legatto, 2011). Studies by Tandang and Arif (2019) have shown that students commonly use metacognitive and social strategies when learning a L2 in the classroom. Moreover, previous research has identified language learning self-efficacy (LLSE), which refers to learners’ confidence in their ability to learn and communicate effectively in the L2 (Campana, 2016), as a crucial factor in successful language learning (Xu et al., 2022). Given these close associations, further research is needed to understand the specific role of metacognitive strategies and LLSE in L2 WTC. This study will, for the first time, explore the growth language mindset and its impact on L2 WTC in Chinese L2 (English) learners and the mechanisms underlying this association with the chain mediating effects of metacognitive strategies and LLSE. The potential relationships among L2 WTC, metacognitive strategies, LLSE, and L2 WTC will be introduced in detail below.

### 1.1. The Relationship/Network Between Language Mindset and L2 WTC

Based on the theory of growth mindset proposed by Dweck (2006, 2013), Ryan and Mercer (2012a, 2012b) introduced the concept of language mindset, which pertains to one’s beliefs and attitudes regarding their own language learning abilities. As mindset can penetrate into specific domains, numerous studies have investigated the role of language mindsets in various aspects of L2 learning (e.g., Khajavy et al., 2021; Papi et al., 2019; Lou et al., 2022). These studies have consistently shown that a growth language mindset, characterized by the belief that language abilities can be developed through effort and practice, is associated with more positive learning outcomes compared to a fixed mindset, which assumes that language abilities are innate and unchangeable. Given the importance of L2 WTC in language learning, it is crucial to consider how variations in mindsets can influence differences in L2 WTC.

Previous studies have indicated that language learners with a growth mindset are more likely to participate in communicative activities, such as speaking and writing, even if they make mistakes (Hejazi et al., 2023). This is because they view language learning as a developmental process and believe that feedback and effort can lead to improvement. Conversely, learners with a fixed mindset may be reluctant to communicate in English due to concerns about being judged or criticized for their language proficiency (Zarrinabadi et al., 2021), which can result in lower levels of willingness to communicate and impede language learning progress. Despite the observed connection between language mindset and L2 WTC, researchers have not fully understood their internal connections, such as their network-level interactions. Willingness to communicate (WTC) is a critical component of language learning, as it directly influences learners’ engagement in communicative activities and their overall language proficiency (MacIntyre et al., 1998). Recent studies have highlighted the importance of WTC in L2 learning, suggesting that it is influenced by a combination of factors, including learners’ attitudes, self-efficacy, and motivation (Dewaele & Dewaele, 2018). A growth language mindset can enhance WTC by fostering a positive attitude towards language learning and encouraging learners to view challenges as opportunities for growth. For example, learners with a growth mindset are more likely to seek feedback and engage in reflective practices, which can improve their language skills and increase their confidence in communicative situations (Lou & Noels, 2017). Additionally, a growth mindset can promote the use of metacognitive strategies, such as goal setting, self-monitoring, and self-assessment, which are known to enhance language learning outcomes (Zarrinabadi et al., 2021). These strategies can empower learners to take control of their learning process, thereby increasing their autonomy and competence in using the L2. In summary, the link between L2 learning and WTC is mediated by learners’ language mindset, which influences their attitudes, strategies, and overall engagement in language learning activities. Future research should further explore the dynamic interplay between these variables to provide a more comprehensive understanding of how mindset influences L2 WTC and language learning outcomes. Hypothesis 1 (H1) posits that learners with a growth language mindset (GLM) exhibit higher levels of L2 willingness to communicate (WTC), as their belief in malleable language abilities fosters proactive engagement in communication despite challenges.

**H1.**  *GLM is positively related to L2 WTC.*

Network analysis is a powerful tool for examining complex systems and associations between variables (Li et al., 2022). In psychological research, this approach can investigate the interconnectedness of psychological constructs (Letina et al., 2019). By using network analysis, researchers can identify significant nodes in a system and gain insights into how modifications to one node can impact others (Schmittmann et al., 2013). In addition to traditional statistical methods, network analysis is a powerful tool for examining complex systems and associations between variables (Li et al., 2022). In psychological research, this approach may investigate the interconnectedness of psychological constructs (Letina et al., 2019). By using network analysis, researchers can identify significant nodes in a system and gain insights into how modifications to one node can impact others (Schmittmann et al., 2013). It has identified certain items, referred to as “bridge items”, which exhibit high centrality within the network. These items serve as pivotal points that connect different parts of the network. This study employs network analysis to explore the complex interrelationships between growth language mindset and L2 WTC. By employing this analysis, we not only gain a more precise understanding of the factors influencing L2 WTC but also provide targeted suggestions for educational practice, such as strengthening students’ communication willingness by emphasizing items identified as bridges in the network analysis. Therefore, network analysis holds significant theoretical and practical importance for uncovering and leveraging these complex relationships to improve language teaching and learning.

### 1.2. The Relationship Among Growth Language Mindset, Metacognitive Strategies, LLSE, and L2 WTC

The relationship between growth language mindset and L2 WTC may entail more intricate internal mechanisms than expected, with potential mediating variables such as metacognitive strategies and LLSE coming into play. According to Oxford (2016), learning strategies encompass how students deliberately and purposefully manage and enhance their learning through cognitive processes. Metacognitive strategies, involving planning, monitoring, and evaluating language learning, stand as a vital repertoire for successful L2 acquisition. The effective deployment of metacognitive strategies can lead to heightened language proficiency and a deeper comprehension of the language (Rahimi & Katal, 2012). These strategies are closely intertwined with mindset in the realm of learning.

Research has also unveiled the significant impacts of metacognitive strategies and LLSE on L2 WTC. Enhanced self-assurance and confidence in language skills prompt learners to initiate communication and tackle more complex communicative tasks (Solhi & Thumvichit, 2024). The utilization of metacognitive strategies can shape learners’ self-efficacy, fostering a sense of accomplishment and motivation that can uplift their self-efficacy. Besides, learners proficient in employing effective learning strategies like planning, reflecting, and monitoring can better evaluate the efficacy of their efforts, thus further boosting their self-efficacy (Teng et al., 2023). Additionally, learners with higher levels of LLSE are more inclined to engage in L2 WTC (Zadorozhnyy & Lee, 2023). Therefore, a possible chain-mediated relationship may exist encompassing growth language mindset, metacognitive strategies, LLSE, and L2 WTC.

Moreover, according to Self-Determination Theory (SDT; Deci & Ryan, 2012), individuals are primarily motivated by intrinsic factors such as autonomy, competence, and relatedness rather than external rewards. Previous studies have highlighted that language mindset can impact intrinsic motivation (Lou & Noels, 2019), with a growth mindset fostering a belief in potential improvement, thereby possibly enhancing intrinsic motivation to communicate in the L2. Additionally, research has shown a link between metacognitive strategies, self-efficacy, and motivation (e.g., Schunk & DiBenedetto, 2021). That is, metacognitive strategies and LLSE would act as mechanisms through which individuals can satisfy their fundamental psychological needs for competence and autonomy, which could influence their willingness to participate in communicative tasks. Hence, from a motivational standpoint at a theoretical level, it is hypothesized that there exists a chain-mediation effect of metacognitive strategies and LLSE on the relationship between growth language mindset and L2 WTC. The second aim of this study is to investigate this hypothesis (H2).

**H2.**  *The relationship between GLM and L2 WTC is sequentially mediated by metacognitive strategies and LLSE.*

### 1.3. The Present Study

**Figure 1:**
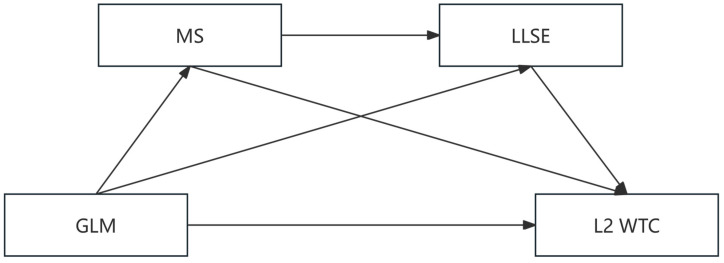
The proposed chain mediation model of GLM and L2 WTC with MS and LLSE as mediators. Note: GLM = growth language mindset, MS = metacognitive strategies, LLSE = language learning self-efficacy, WTC = willingness to communicate.

This study aimed to address two hypotheses to elucidate the mechanisms linking growth language mindset (GLM) and L2 willingness to communicate (L2 WTC). Hypothesis 1 (H1) postulated a direct positive association between GLM and L2 WTC, predicated on the theoretical premise that learners endorsing a belief in the malleability of language abilities exhibit greater propensity to engage in L2 communication. Hypothesis 2 (H2) extended this framework by proposing a sequential chain mediation model (see Figure 1), wherein GLM indirectly enhances L2 WTC through the intermediary roles of metacognitive strategies (MS) and language learning self-efficacy (LLSE). Grounded in Self-Determination Theory (SDT; Deci & Ryan, 2012)—which underscores intrinsic motivation, competence, and autonomy as catalysts for goal-directed behavior—and the Strategic Self-Regulation Model (SSR; Oxford, 2016), this investigation posits that GLM fosters adaptive self-regulatory practices (e.g., goal-setting, progress monitoring) and bolsters learners’ confidence in their linguistic capabilities, thereby amplifying communicative engagement. Methodologically, the study combined network analysis to map the structural interplay between GLM and L2 WTC at the item level, alongside chain mediation analysis, to quantify the indirect pathways theorized in H2. By synthesizing these approaches, the research aimed to advance a holistic understanding of how mindset-driven cognition and strategic behaviors converge to shape communicative outcomes in L2 contexts, offering both theoretical and pedagogical implications for fostering resilience and agency in language learners.

## 2. Materials

### 2.1. Participants

A total of 532 Chinese L2 (English) learners in Guangdong Province (China) participated in the study. The participants primarily speak Mandarin Chinese and their major is related to education. With 524 valid questionnaires collected, the response rate was 98.50%. The participants’ age ranged from 17 to 25 years (*M* ± *SD* = 20.42 ± 1.29 years). Out of the participants, 171 (32.60%) were male and 353 (67.40%) were female. All participants were native Chinese speakers who had passed at least one of the English proficiency exams, such as TEM-4 or TEM-8 (Test for English Majors-Band 4/8), or the Chinese college entrance exams (Gao Kao), based on their self-reported information regarding the exams they had passed.

### 2.2. Procedure

The research study received ethical approval from the Human Research Ethics Committee for Non-Clinical Faculties at the School of Psychology. Participants were recruited via convenience sampling through university mailing lists, social media groups, and online forums targeting English learners in China (e.g., students enrolled in language programs or members of language exchange communities). After providing informed consent—which included a detailed explanation of the study’s purpose, voluntary participation, and data anonymization procedures—participants completed a battery of self-report questionnaires hosted on WJX (https://www.wjx.cn/; accessed on 10 April 2025), a Chinese survey platform akin to Qualtrics. WJX supports Mandarin interfaces, data encryption, and adaptive question formats (e.g., Likert scales, open-ended prompts) while anonymizing responses to exclude identifiers like IP addresses. This design aligned with China’s data privacy standards and the ethics committee’s confidentiality requirements.

### 2.3. Tools

#### 2.3.1. Growth Language Mindset

The Language Mindsets Inventory (LMI), developed by Lou and Noels (2017), was utilized to assess individuals’ language mindsets, comprising 9 items measuring growth mindset and 9 for fixed mindset. The study employed the growth mindset subscale to evaluate L2 students’ growth mindsets across three subdimensions: general language intelligence beliefs (3 items: e.g., “No matter who you are, you can significantly improve your English abilities.”), L2 aptitude beliefs (3 items: e.g., “You can always improve your English ability.”), and age sensitivity beliefs (3 items: e.g., “No matter how old you are, you can learn English well as long as they work hard.”) about language learning. To ensure relevance to the study context, the original questionnaire’s references to “L2” were replaced with “English”. Participants rated each item on a 6-point scale ranging from 1 (strongly disagree) to 6 (strongly agree). Higher mean scores reflected stronger growth language mindsets. The growth language mindset measure demonstrated high internal consistency reliability for the current study, as indicated by a Cronbach’s alpha of 0.89.

#### 2.3.2. Metacognitive Strategies

The measurement of metacognitive strategies in this study utilized a scale adapted by Wong (2002) from Bachman et al.’s (1993) metacognitive strategies questionnaire. The scale consisted of 27 items and was assessed on a 5-point scale ranging from 1 (never) to 5 (always). It aimed to assess planning (13 items: e.g., “Before using English, I always consider whether my grammar is sufficient.”), monitoring (8 items: e.g., “When speaking English, I am aware of which words I pronounce incorrectly.”), and evaluating (6 items: e.g., “I reflect on whether I have made progress in my English learning.”). Higher mean scores indicated stronger metacognitive abilities. The overall scale demonstrated a Cronbach’s alpha of 0.95, while the subscales of planning, monitoring, and evaluating exhibited Cronbach’s alpha values of 0.89, 0.84, and 0.87.

#### 2.3.3. Language Learning Self-Efficacy

Language learning self-efficacy was assessed by the Questionnaire of English Self-efficacy (QESE), developed by C. Wang et al. (2014). The scale consists of 32 items rated on a 7-point scale ranging from 1 (I cannot do it at all) to 7 (I can do it very well). The QESE is designed to measure self-efficacy in four domains: listening (8 items: e.g., “Can you understand stories told in English?”), speaking (8 items: e.g., “Can you describe your university to other people in English?”), reading (8 items: e.g., “Can you read English news on the internet?”), and writing (8 items: e.g., “Can you write email messages in English?”). Each domain assesses confidence in specific tasks, such as understanding stories in English or introducing one’s university in English. Higher mean scores indicating higher level of language learning self-efficacy. The internal consistency of responses was acceptable, as evidenced by a Cronbach’s alpha of 0.98.

#### 2.3.4. L2 WTC

The items measuring L2 WTC were adapted from Lee and Hsieh (2019) and used to assess individuals’ L2 WTC in the classroom (5 items; e.g., “When you have an opportunity to make a presentation in front of a large group”) and L2 WTC in extramural digital contexts (5 items; e.g., “When you have an opportunity to explain your own culture online in English to other English speakers.”). These 10 items were assessed using a 5-point Likert scale, ranging from 1 (definitely not willing) to 5 (definitely willing). The overall scale showed a Cronbach’s alpha of 0.94.

## 3. Analysis

The measurement instruments in this study utilized varied response formats (e.g., 6-point Likert scale for Growth Language Mindset, 5-point for Metacognitive Strategies and L2 WTC, 7-point for LLSE). To facilitate scale comparability in subsequent analyses, raw scores were statistically standardized into z-scores prior to modeling. A rigorous data preprocessing protocol ensured data integrity through multiple measures: Missing data were addressed through listwise deletion for cases exceeding 10% incomplete responses. Outliers were identified via boxplot analysis and Z-score thresholds (|Z| > 3), with extreme values replaced by variable-specific medians or means. Logical consistency checks were implemented by cross-verifying responses between theoretically linked constructs (e.g., growth mindset and metacognitive strategy items). Psychometric evaluations confirmed strong reliability, with Cronbach’s α coefficients indicating high internal consistency (growth language mindset: α = 0.89; metacognitive strategies: α = 0.95; language learning self-efficacy: α = 0.98; L2 WTC: α = 0.94). Test–retest reliability over a two-week interval showed strong temporal stability for key constructs (growth mindset: r = 0.76, *p* < 0.001; self-efficacy: r = 0.81, *p* < 0.001). Potential common method bias was assessed through Harman’s single-factor test, confirming that common method variance did not unduly influence the results. These comprehensive procedures ensured robust data quality for subsequent analytical modeling.

Subsequently, a network analysis was performed to visualize the internal connections between growth language mindset and L2 WTC. This analysis utilized the Extended Bayesian Information Criterion graphical least absolute shrinkage and selection operator (EBICglasso) implemented in the R-package (version 4.3.1) qgraph (version 1.9.5) (Epskamp et al., 2012). Centrality indices of strength were calculated using the centrality Plot function within the qgraph R-package (Opsahl et al., 2010). Edge-weight accuracy and centrality stability were computed separately using the R-packages networktools (version 1.5.0) and boonet (version 1.5.1) (Epskamp et al., 2018). Supplementary Materials included additional figures for reference.

In addition, we conducted correlation and mediation analyses to investigate the mediating effects of metacognitive strategies and LLSE between growth language mindset and L2 WTC. Descriptive statistics and Pearson correlations were computed using SPSS 26.0 to examine the means, standard deviations, and associations among the observed variables. For the multiple mediation analysis, we utilized the SPSS macro PROCESS (Model 6) with a 95% confidence interval (CI) based on 5000 bootstrap samples, as recommended by Hayes (2015) (available at www.processmacro.org; accessed on 10 April 2025).

## 4. Results

### 4.1. Analysis of Common Method Bias

To assess common method bias, the Harman single factor test (Podsakoff et al., 2003) was conducted, wherein any factor accounting for over 50% of the total variance would indicate a potential issue (Podsakoff & Organ, 1986). In this study, the eigenvalues of the 9 factors exceeded 1, with the highest eigenvalue explaining only 41.55% of the total variance. Consequently, no significant common method bias was observed.

### 4.2. Descriptive Statistics and Correlations

Table 1 presents the descriptive statistics including means (*M*), standard deviations (*SD*), skewness, and kurtosis of all variables measured among Chinese university students. The results indicate significant correlations between all variables (*p*s < 0.05), consistent with conceptual expectations. Specifically, growth language mindset exhibits positive correlations with metacognitive strategies, language learning self-efficacy, and L2 WTC. Furthermore, metacognitive strategies are positively correlated with LLSE and L2 WTC, while LLSE shows a positive correlation with L2 WTC. Given that gender, age, and frequency of use for English learning apps demonstrated a significant association with at least one of the target variables (*p*s < 0.05), they were incorporated as control variables in the subsequent mediation analyses. Additionally, gender was included as a control variable also due to the strong imbalance of gender distribution in our sample.

### 4.3. Network Analysis of Language Mindset and L2 WTC

**Figure 2:**
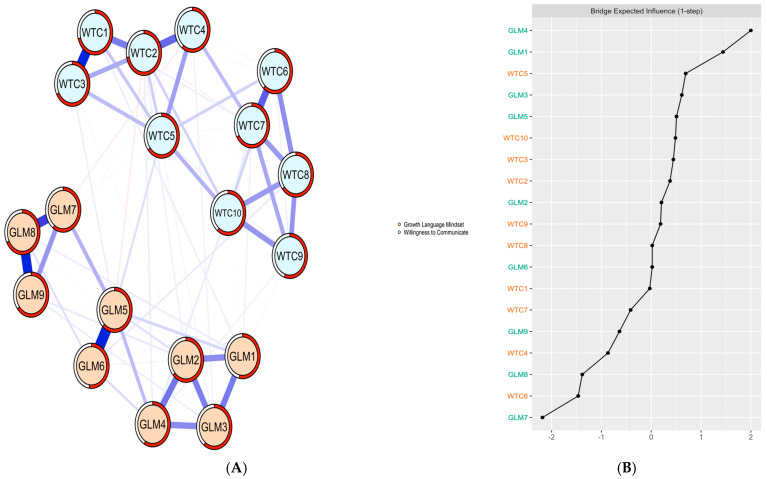
EBICglasso model based on the item-level: (**A**) bridge centrality: Bridge Expected Influence (BEI); (**B**) network analysis according to the relationships between GLM and L2 WTC among 524 participants. Note: GLM = growth language mindset, WTC = willingness to communicate. The thickness of edges reflected the strength of associations among items; the blue and orange edges signify positive and negative correlations, respectively.

Figure 2A presents the EBICglasso network, which explores the relationship between growth language mindset and willingness to communicate in L2 among 524 Chinese university students. The network consists of 19 nodes and 82 non-zero edges. The analysis revealed a positive correlation between growth language mindset and willingness to communicate in L2. Figure 2B shows that the bootstrapped confidence intervals of edge-weight parameters demonstrate the reliability of the current network structure. Additionally, the case-dropping bootstrap procedure indicated that the EI value (0.59) was preferable in the network (*CS-C* ≥ 0.50). Furthermore, the BEI value (0.36) was considered acceptable in the network (*CS-C* ≥ 0.25). Notably, items GLM4 (item 4 of growth language mindset; 2.00; i.e., “You can always change your L2 ability”) exhibited higher levels of bridge EI centrality.

### 4.4. The Chain Mediation Effects Analyses

The chain mediation model was constructed to examine the relationships among growth language mindset (GLM), metacognitive strategies (MS), language learning self-efficacy (LLSE), and willingness to communicate in L2 (L2 WTC). The model included one direct path (GLM → L2 WTC) and three indirect paths (GLM → MS → L2 WTC; GLM → LLSE → L2 WTC; GLM → MS → LLSE → L2 WTC). Covariates such as age, gender, and FUELA were considered to ensure conservative predictions. The results of the chain mediation analysis, conducted using the PROCESS macro v 3.0 for SPSS 26.0 software, are summarized in Table 2. GLM significantly predicted MS (*β* = 0.4875, *t* = 13.07, *p* < 0.001), and GLM was positively correlated with LLSE (*β* = 0.2855, *t* = 8.37, *p* < 0.001). MS exhibited a positive correlation with LLSE (*β* = 0.5291, *t* = 15.12, *p* < 0.001). Furthermore, GLM significantly predicted L2 WTC (*β* = 0.0808, *t* = 2.21, *p* < 0.05), LLSE significantly predicted L2 WTC (*β* = 0.6060, *t* = 13.68, *p* < 0.001), and MS had a significant positive prediction effect on L2 WTC (*β* = 0.1079, *t* = 2.56, *p* < 0.05). The results indicated that GLM had a significant positive predictive effect on L2 WTC (*β* = 0.46, *p* < 0.001). Specifically, the direct effect of GLM on L2 WTC was significant (*β* = 0.08 *p* < 0.05), while the total indirect effect through MS and LLSE was substantial (*β* = 0.38, *p* < 0.001). This suggests that the chain mediation of MS and LLSE accounted for a meaningful portion of the relationship between GLM and L2 WTC.

**Figure 3:**
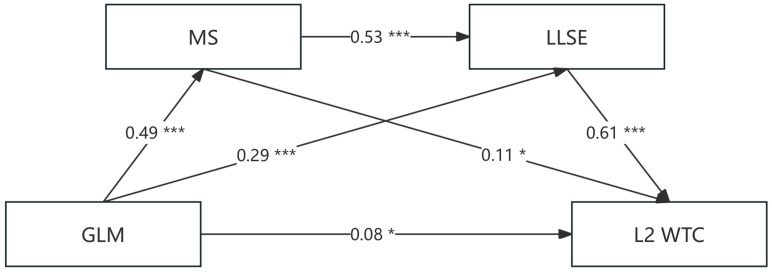
The chain mediation model. Note: * *p* < 0.05, *** *p* < 0.001.

To assess the significance of the mediation effects in the chain mediation model, the bootstrapping method (5000 resamples) was conducted using Hayes’ PROCESS macro (Model 6) on a sample of 524 participants, controlling for age, gender, and frequency of English learning app use. The results, presented in Table 2 and Table 3, revealed a significant direct effect of growth language mindset (GLM) on L2 willingness to communicate (L2 WTC) (*β* = 0.08, SE = 0.04, 95% CI [0.01, 0.15]). Additionally, three indirect pathways were identified: (1) GLM → metacognitive strategies (MS) → L2 WTC (*β* = 0.05, SE = 0.03, 95% CI [0.003, 0.11]), (2) GLM → language learning self-efficacy (LLSE) → L2 WTC (*β* = 0.17, SE = 0.03, 95% CI [0.11, 0.24]), and (3) the sequential chain GLM → MS → LLSE → L2 WTC (*β* = 0.16, SE = 0.02, 95% CI [0.12, 0.20]). The total indirect effect accounted for 82.6% of the total effect (*β* = 0.46, *p* < 0.001), with LLSE emerging as the strongest mediator (*β* = 0.61, *p* < 0.001). The model explained 56% of the variance in L2 WTC (*R*^2^ = 0.56), aligning with the Strategic Self-Regulation Model’s emphasis on metacognitive strategies and LLSE as key drivers of communicative confidence (see Figure 3). These findings demonstrate that GLM exerts its influence both directly and via a sequential chain, wherein metacognitive strategies enhance language learning self-efficacy, which in turn bolsters L2 WTC.

## 5. Discussion

This study provides valuable insights into the practical significance of growth language mindset, metacognitive strategies, and LLSE in predicting L2 WTC. The use of network analysis has revealed key indicators that link growth language mindset and L2 WTC, such as items GLM4 and GLM1. These indicators are critical in facilitating the relationship between the growth language mindset and the willingness to communicate in L2. The chain mediation model further highlights that metacognitive strategies and LLSE play crucial roles in enhancing L2 WTC. Specifically, the model explains 85% of the variance in L2 WTC, with LLSE (*β* = 0.61) having the largest practical impact. These findings suggest that fostering a growth language mindset and enhancing metacognitive strategies and LLSE can lead to meaningful improvements in learners’ willingness to communicate in a L2.

### 5.1. The Effect of Growth Language Mindset on L2 WTC

This study provides compelling evidence on the meaningful impact of growth language mindset, metacognitive strategies, and LLSE on L2 WTC. The findings emphasize the practical significance of fostering a growth-oriented mindset and enhancing learners’ self-efficacy to improve their willingness to communicate in a L2. The chain mediation model, which explains 85% of the variance in L2 WTC, underscores the importance of these factors in language education. By focusing on these elements, educators can create more effective learning environments that support long-term language development and communicative competence.

In addition, utilizing network analysis in this study has been instrumental in identifying three key bridge indicators at the item level: GLM4, GLM1, and WTC5. These bridge items are pivotal as they serve as conduits that enhance the relationship between a growth language mindset and L2 WTC. According to Self-Determination Theory (SDT; Deci & Ryan, 2012), a growth language mindset enhances intrinsic motivation by fulfilling psychological needs for competence and autonomy. This mindset nurtures the belief in one’s potential for linguistic improvement and proficiency development, as noted by Fathi et al. (2024), which is especially pertinent for L2 learners facing the challenges of effective communication in a L2. Embracing a robust growth language mindset can significantly boost L2 learners’ motivation to overcome these challenges, as suggested by Lou and Noels (2020), thereby increasing their natural inclination to engage in L2 communication. This intrinsic motivation is further reinforced by the pursuit of competence and autonomy. Individuals with a growth mindset are more likely to perceive obstacles as opportunities for learning and skill enhancement, as indicated by Zarrinabadi et al. (2021), approaching communicative tasks with enthusiasm and determination, which in turn enhances their readiness to engage in L2 communication.

The network analysis has provided a deeper understanding of the intricate relationship between a growth language mindset and L2 WTC, building upon existing research that highlights the influence of students’ beliefs on WTC. The study particularly emphasizes the importance of key bridge indicators, such as GLM4 (“You can always change your L2 ability”), GLM1 (“No matter who you are, you can significantly change your language intelligence level”), and WTC5 (“When you have an opportunity to explain in English your own culture to your classmates”). These indicators are crucial as they underscore the necessity of fostering a belief in one’s ability to improve language skills continuously, which is essential for motivating and empowering learners to actively participate in L2 communication. It reveals that individuals with a growth-oriented language mindset do not view language as a barrier but rather as a facilitator for connection and communication. They regard the acquisition and use of a L2 as a tool for expressing thoughts, emotions, and cultural heritage, thus emphasizing language’s role as an enabler of expression rather than a limitation, as further supported by Zhong et al. (2023). The identification of these bridge items through network analysis is not merely an academic exercise; it has practical implications for educators and language learning practitioners. By focusing on these specific items, educators can design targeted interventions that cultivate a growth mindset and enhance learners’ confidence in their language abilities. This targeted approach can lead to more effective strategies for improving L2 WTC, making the findings from network analysis a valuable tool for both research and practice.

### 5.2. The Mediating Roles of Metacognitive Strategies and LLSE

The current study has identified the significant mediating roles of metacognitive strategies and LLSE in the association between growth language mindset and L2 WTC. These mediators operate both separately and sequentially. The findings reveal that metacognitive strategies and LLSE not only mediate the relationship between growth language mindset and L2 WTC but also contribute significantly to the overall effect size. The chain mediation effect (*β* = 0.38, *p* < 0.001) indicates that fostering metacognitive strategies and enhancing LLSE can meaningfully amplify the impact of a growth language mindset on L2 WTC. This suggests practical significance beyond mere statistical significance, as these factors can be targeted in educational interventions to improve language learning outcomes on the link between growth mindset and metacognitive strategies (Zarrinabadi et al., 2021), as well as the connection between metacognitive strategies and L2 WTC. A positive growth language mindset may promote a growth-oriented attitude, resilience, and a willingness to take risks in using the L2 (Lamb, 2017).

Furthermore, this observation resonates with the Strategic Self-Regulation (SSR) Model advanced by Oxford (2016), which underscores the pivotal role of self-regulation in language acquisition. This model entails planning, monitoring, and evaluating one’s learning processes, empowering learners to seize control of their learning journey and augment their LLSE (Teng et al., 2023). Consequently, metacognitive strategies and LLSE could also be intricately intertwined within the language learning domain. The metacognitive strategies could enable learners to set clear goals, track their progress, and adjust their approaches as needed. As a result, their self-efficacy and confidence in using the L2 increase, making them more willing to communicate.

### 5.3. Implications

The findings of this study offer actionable insights for educators, teacher trainers, and policymakers to collaboratively enhance language education. For educators, fostering a growth language mindset can be achieved by reframing challenges as learning opportunities—for instance, facilitating peer error-analysis sessions or providing feedback that emphasizes effort. Structured metacognitive training, such as weekly SMART goal-setting templates for vocabulary acquisition or self-assessment checklists, can empower students to take ownership of their learning. Low-stakes practice environments, like role-plays with peer feedback or digital language journals (e.g., Flipgrid video diaries), further build confidence through incremental successes. Teacher trainers can amplify these efforts by modeling growth mindset pedagogy in professional development workshops, such as demonstrating how to frame corrective feedback as “next steps” rather than failures, and by equipping educators with tools like guided reflection prompts. Policymakers, in turn, can drive systemic change by mandating growth mindset and metacognitive modules in national language standards, funding AI-driven tutoring platforms with real-time self-assessment features, and launching public campaigns to reframe language proficiency as a developable skill rather than an innate talent. Together, these strategies create an ecosystem where resilient, self-directed learners thrive, supported by curricula, tools, and societal attitudes that prioritize effort and adaptability over perfection.

### 5.4. Limitations and Further Studies

Our study offers new evidence on the link between growth language mindset and L2 WTC, but there are limitations to consider. Firstly, our research focused on a specific group of Chinese L2 (English) learners, which may limit the applicability of the findings to other cultural and linguistic contexts. Secondly, relying on self-reported data in our study may introduce potential biases and subjectivity. Additionally, the cross-sectional nature of the study limits our ability to establish causal relationships among the variables. Future research could address these limitations by conducting longitudinal studies with diverse participant groups to better understand the long-term effects of growth language mindset on L2 WTC. Qualitative studies could provide deeper insights into the subjective experiences and perceptions of individuals with a growth language mindset. Furthermore, intervention studies could explore the effectiveness of targeted interventions aimed at fostering growth language mindsets and their impact on L2 WTC. These avenues for future research would contribute to a more comprehensive understanding of the role of growth language mindsets in language learning and communication.

## 6. Conclusions

The aims of this study substantiate both hypotheses, establishing a significant positive association between a growth language mindset (GLM) and L2 willingness to communicate (L2 WTC; H1) and elucidating the sequential mediation of this relationship through metacognitive strategies (MS) and language learning self-efficacy (LLSE; H2). The chain mediation model accounted for 85% of the variance in L2 WTC, with LLSE (*β* = 0.61) demonstrating the most pronounced mediating influence, underscoring its critical role in fortifying learners’ confidence to engage in L2 communication. Network analysis further delineated pivotal bridge indicators (e.g., GLM4, GLM1, WTC5), which illuminate actionable pathways for reinforcing adaptive beliefs and communicative behaviors. These results align with theoretical frameworks such as Self-Determination Theory, which posits that intrinsic motivation arises from fulfilling psychological needs for competence and autonomy, and the Strategic Self-Regulation Model, which emphasizes the centrality of planning, monitoring, and evaluative strategies in language acquisition. Collectively, the study underscores the imperative of integrating growth mindset cultivation, explicit metacognitive strategy instruction, and self-efficacy-enhancing practices within pedagogical frameworks. Such interventions hold transformative potential for fostering resilient, self-regulated L2 learners capable of navigating communicative challenges with proactive engagement. These insights advocate for systemic curricular reforms and educator training initiatives to prioritize psychological and strategic dimensions of language learning, thereby advancing both theoretical and practical paradigms in SLA research.

## Figures and Tables

**Table 1:** Descriptive statistics and Pearson correlations among all variables.

Variables	*M*	*SD*	1	2	3	4	5	6	7
1. Gender ^a^	-	-	-						
2. Age	20.42	1.29	−0.18 ***	-					
3. FUELA ^b^	4.17	0.78	−0.13 **	0.04	-				
4. GLM	4.46	0.74	−0.04	0.01	0.12 ***	-			
5. MS	3.45	0.61	−0.15 ***	−0.009	0.24 ***	0.52 ***	-		
6. LLSE	4.79	0.97	−0.17 ***	0.01 *	0.24 ***	0.57 ***	0.70 ***	-	
7. L2 WTC	3.43	0.77	−0.16 ***	−0.42 **	0.18 ***	0.49 ***	0.57 ***	0.76 ***	-
Skewness (<±2)			-	-	-	−0.38	−0.03	−0.32	−0.06
Kurtosis (<±7)			-	-	-	0.21	0.12	0.12	−0.17

Note. N = 524. ^a^ 0 = male, 1 = female, ^b^ Frequency of use for English learning apps = FUELA, 1 = never use, 2 = a few times a month, 3 = several time a week, 4 = nearly every day, 5 = every day. * *p* < 0.05, ** *p* < 0.01, *** *p* < 0.001.

**Table 2:** The chain mediation model of GLM and MS between LLSE and L2 WTC.

Variables	MS				LLSE				L2 WTC			
	*β*	95% CI	SE	*t*	*β*	95% CI	SE	*t*	*β*	95% CI	SE	*t*
Gender	−0.1254	[−0.42, −0.11]	0.08	−3.34 ***	−0.0507	[−0.23, 0.02]	0.06	−1.69	−0.0250	[−0.18, 0.07]	0.07	−0.82
Age	−0.0409	[−0.42, 0.025]	0.03	−1.10	0.1011	[0.03, 0.12]	0.02	3.42 ***	0.0642	[0.004, 0.10]	0.02	2.13 *
FUELA	0.1327	[0.08, 0.27]	0.05	3.53 ***	0.0497	[−0.01, 0.14]	0.04	1.65	−0.0141	[−0.09, 0.06]	0.04	−0.46
GLM	0.4875	[0.41, 0.56]	0.04	13.07 ***	0.2855	[0.22, 0.35]	0.03	8.37 ***	0.0808	[0.09, 0.15]	0.04	2.21 *
MS					0.5291	[0.46, 0.60]	0.04	15.12 ***	0.1079	[0.03, 0.19]	0.04	2.56 *
LLSE									0.6060	[0.52, 0.69]	0.04	13.68 ***
*R* ^2^	0.30				0.56				0.56			
*F*	56.43 ***				133.16 ***				107.96 ***			

Note. N = 524, *β* = standardized coefficient. * *p* < 0.05, *** *p* < 0.001.

**Table 3:** The chain mediation analysis of GLM on L2 WTC.

Mediating Effects	Effects	Boot SE	Boot LLCI	Boot ULCI
Total effect	0.46	0.04	0.39	0.54
Direct effect	0.08	0.04	0.01	0.15
Total indirect effects	0.38	0.04	0.31	0.46
Ind1: X → M_1_ → Y	0.05	0.03	0.003	0.11
Ind2: X → M_2_ → Y	0.17	0.03	0.11	0.24
Ind3: X → M_1_ → M_2_ → Y	0.16	0.02	0.12	0.20

Note. Ind = indirect path. X = GLM, M_1_ = MS. M_2_ = LLSE. Y = L2 WTC. Bootstrap sample size = 5000. Boot SE = bootstrap standard error; Boot LLCI = bootstrap low limit of confidence interval; Boot ULCI = bootstrap upper limit of confidence interval.

## Data Availability

The raw data supporting the conclusions of this article will be made available by the authors, without undue reservation.

## Supplementary Materials

The following supporting information can be downloaded at: https://www.mdpi.com/article/10.3390/bs15040521/s1, S1: Growth language mindset inventory; S2: Metacognitive strategies questionnaire; S3: Language learning self-efficacy questionnaire; S4: L2 WTC questionnaire.
