# Supplementary material for: The Roles of Growth Language Mindset, Metacognitive Strategies, and Language Learning Self-Efficacy in Predicting L2 Willingness to Communicate: A Network Analysis and a Chain Mediation Model
Source: Behav Sci (Basel). 2025 Apr 13;15(4):521. doi: 10.3390/bs15040521 (PMC12024291; doi:10.3390/bs15040521)
Supplement: Supplementary file 1 [file behavsci-15-00521-s001.zip › behavsci-3497617-supplementary.pdf]

Supplementary Materials

S1. Growth language mindset Inventory

In this section, you will read nine statements. Please indicate whether you agree or disagree with the views expressed in these statements. You have six options: 1 (Strongly Disagree), 2 (Moderately Disagree), 3 (Slightly Disagree), 4 (Slightly Agree), 5 (Moderately Agree), and 6 (Strongly Agree). There are no right or wrong answers for these options, and you do not need to spend too much time thinking about any single item.					
GM1	No matter who you are, you can significantly improve your English abilities.				
1<br>Strongly<br>Disagree	2<br>Moderately<br>Disagree	3<br>Slightly<br>Disagree	4<br>Slightly<br>Agree	5<br>Moderately<br>Agree	6<br>Strongly<br>Agree
GM2	You can always substantially improve your English intelligence.				
1<br>Strongly<br>Disagree	2<br>Moderately<br>Disagree	3<br>Slightly<br>Disagree	4<br>Slightly<br>Agree	5<br>Moderately<br>Agree	6<br>Strongly<br>Agree
GM3	No matter how much English intelligence you have, you can always improve it quite a bit.				
1<br>Strongly<br>Disagree	2<br>Moderately<br>Disagree	3<br>Slightly<br>Disagree	4<br>Slightly<br>Agree	5<br>Moderately<br>Agree	6<br>Strongly<br>Agree
GM4	You can always improve your English ability.				
1<br>Strongly<br>Disagree	2<br>Moderately<br>Disagree	3<br>Slightly<br>Disagree	4<br>Slightly<br>Agree	5<br>Moderately<br>Agree	6<br>Strongly<br>Agree
GM5	In learning English, if you work hard at it, you will always get better.				
1<br>Strongly<br>Disagree	2<br>Moderately<br>Disagree	3<br>Slightly<br>Disagree	4<br>Slightly<br>Agree	5<br>Moderately<br>Agree	6<br>Strongly<br>Agree
GM6	How good you are at using English will always improve if you really work at it.				
1<br>Strongly<br>Disagree	2<br>Moderately<br>Disagree	3<br>Slightly<br>Disagree	4<br>Slightly<br>Agree	5<br>Moderately<br>Agree	6<br>Strongly<br>Agree
GM7	No matter how old you are, you can learn English well as long as they work hard.				
1<br>Strongly<br>Disagree	2<br>Moderately<br>Disagree	3<br>Slightly<br>Disagree	4<br>Slightly<br>Agree	5<br>Moderately<br>Agree	6<br>Strongly<br>Agree
GM8	How well a person learns English does not depend on age: anyone who works hard can be a fluent speaker in that language.				
1<br>Strongly<br>Disagree	2<br>Moderately<br>Disagree	3<br>Slightly<br>Disagree	4<br>Slightly<br>Agree	5<br>Moderately<br>Agree	6<br>Strongly<br>Agree
GM9	Regardless of the age at which they start, people can learn another language well.				
1<br>Strongly	2<br>Moderately	3<br>Slightly	4<br>Slightly	5<br>Moderately	6<br>Strongly

Disagree	Disagree	Disagree	Agree	Agree	Agree

S2. Metacognitive strategies questionnaire

Please answer the following questions based on your thoughts and practices during the process of learning English. These questions are related to your metacognitive strategies in English learning. Think about your experiences in learning English and then choose the answers that best match your experiences. There are no right or wrong choices.				
1	Before using English, I always consider whether my grammar is sufficient.			
1<br>Never	2<br>Rarely	3<br>Sometimes	4<br>Frequently	5<br>Always
2	In English learning, I make plans to make the best use of my time.			
1<br>Never	2<br>Rarely	3<br>Sometimes	4<br>Frequently	5<br>Always
3	I usually understand the purpose of classroom activities.			
1<br>Never	2<br>Rarely	3<br>Sometimes	4<br>Frequently	5<br>Always
4	I will immediately use the new sentence patterns and new words I have just learned to strengthen my understanding.			
1<br>Never	2<br>Rarely	3<br>Sometimes	4<br>Frequently	5<br>Always
5	When speaking English, I am aware of which words I pronounce incorrectly.			
1<br>Never	2<br>Rarely	3<br>Sometimes	4<br>Frequently	5<br>Always
6	I set goals for my English learning.			
1<br>Never	2<br>Rarely	3<br>Sometimes	4<br>Frequently	5<br>Always
7	I reflect on whether I have made progress in my English learning.			
1<br>Never	2<br>Rarely	3<br>Sometimes	4<br>Frequently	5<br>Always
8	When practicing listening, I know which parts I don't understand.			
1<br>Never	2<br>Rarely	3<br>Sometimes	4<br>Frequently	5<br>Always
9	I know what methods can help me remember new words.			
1<br>Never	2<br>Rarely	3<br>Sometimes	4<br>Frequently	5<br>Always
10	When speaking English, I can realize when I make grammatical mistakes.			
1<br>Never	2<br>Rarely	3<br>Sometimes	4<br>Frequently	5<br>Always
11	I always listen attentively when others speak English.			
1<br>Never	2<br>Rarely	3<br>Sometimes	4<br>Frequently	5<br>Always
12	I test myself on the new words or phrases I have just learned to see if I have remembered them well.			

1<br>Never	2<br>Rarely	3<br>Sometimes	4<br>Frequently	5<br>Always
13	After I finish an English conversation, I think about how I can make it better.			
1<br>Never	2<br>Rarely	3<br>Sometimes	4<br>Frequently	5<br>Always
14	I can hear when others make grammatical mistakes.			
1<br>Never	2<br>Rarely	3<br>Sometimes	4<br>Frequently	5<br>Always
15	When I do homework, I always have a dictionary or other reference books at hand.			
1<br>Never	2<br>Rarely	3<br>Sometimes	4<br>Frequently	5<br>Always
16	Before writing an essay, I always plan how to write it.			
1<br>Never	2<br>Rarely	3<br>Sometimes	4<br>Frequently	5<br>Always
17	I try to create a good learning environment for myself.			
1<br>Never	2<br>Rarely	3<br>Sometimes	4<br>Frequently	5<br>Always
18	I review after class.			
1<br>Never	2<br>Rarely	3<br>Sometimes	4<br>Frequently	5<br>Always
19	I try to create a good learning environment for myself.			
1<br>Never	2<br>Rarely	3<br>Sometimes	4<br>Frequently	5<br>Always
20	I preview before class.			
1<br>Never	2<br>Rarely	3<br>Sometimes	4<br>Frequently	5<br>Always
21	When I decide to study, I cannot be disturbed by the outside.			
1<br>Never	2<br>Rarely	3<br>Sometimes	4<br>Frequently	5<br>Always
22	After I learn a knowledge point, I test myself to see if I have really mastered it.			
1<br>Never	2<br>Rarely	3<br>Sometimes	4<br>Frequently	5<br>Always
23	When I talk with others in English, I pay attention to whether the other party understands my meaning.			
1<br>Never	2<br>Rarely	3<br>Sometimes	4<br>Frequently	5<br>Always
24	When I start studying, I focus all my energy on my study.			
1<br>Never	2<br>Rarely	3<br>Sometimes	4<br>Frequently	5<br>Always
25	When I do some English homework, I think about whether my existing knowledge is enough.			
1<br>Never	2<br>Rarely	3<br>Sometimes	4<br>Frequently	5<br>Always

26	I choose learning methods according to my own characteristics.			
1<br>Never	2<br>Rarely	3<br>Sometimes	4<br>Frequently	5<br>Always
27	I adjust my learning plan according to the actual situation.			
1<br>Never	2<br>Rarely	3<br>Sometimes	4<br>Frequently	5<br>Always

S3. Language learning self-efficacy questionnaire

Please read the following descriptions carefully and accurately evaluate the content described based on your own situation, whether or not you are currently doing these things. These items are designed to measure your judgment of your own English abilities. There are no right or wrong answers. (1 = I cannot do it at alll, 7 = I can do it well)						
1	Can you understand stories told in English?					
1<br>I cannot do it at all	2<br>I cannot do it	3<br>Maybe I cannot do it	4<br>Maybe I can do it	5<br>I basically do it	6<br>I can do it	7<br>I can do it well
2	Can you do homework alone when they include reading English texts?					
1<br>I cannot do it at all	2<br>I cannot do it	3<br>Maybe I cannot do it	4<br>Maybe I can do it	5<br>I basically do it	6<br>I can do it	7<br>I can do it well
3	Can you understand American English TV programs?					
1<br>I cannot do it at all	2<br>I cannot do it	3<br>Maybe I cannot do it	4<br>Maybe I can do it	5<br>I basically do it	6<br>I can do it	7<br>I can do it well
4	Can you describe your university to other people in English?					
1<br>I cannot do it at all	2<br>I cannot do it	3<br>Maybe I cannot do it	4<br>Maybe I can do it	5<br>I basically do it	6<br>I can do it	7<br>I can do it well
5	Can you compose messages in English on the internet (face book, twitter, blogs, etc.)?					
1<br>I cannot do it at all	2<br>I cannot do it	3<br>Maybe I cannot do it	4<br>Maybe I can do it	5<br>I basically do it	6<br>I can do it	7<br>I can do it well
6	Can you describe the way to the university from the place where you live in English?					
1<br>I cannot do it at all	2<br>I cannot do it	3<br>Maybe I cannot do	4<br>Maybe I can do it	5<br>I basically do it	6<br>I can do it	7<br>I can do it well

		it				
7	Can you write English compositions assigned by your English instructor?					
1<br>I cannot do it at all	2<br>I cannot do it	3<br>Maybe I cannot do it	4<br>Maybe I can do it	5<br>I basically do it	6<br>I can do it	7<br>I can do it well
8	Can you tell a story in English?					
1<br>I cannot do it at all	2<br>I cannot do it	3<br>Maybe I cannot do it	4<br>Maybe I can do it	5<br>I basically do it	6<br>I can do it	7<br>I can do it well
9	Can you understand radio programs in English speaking countries?					
1<br>I cannot do it at all	2<br>I cannot do it	3<br>Maybe I cannot do it	4<br>Maybe I can do it	5<br>I basically do it	6<br>I can do it	7<br>I can do it well
10	Can you understand English TV programs made in China?					
1<br>I cannot do it at all	2<br>I cannot do it	3<br>Maybe I cannot do it	4<br>Maybe I can do it	5<br>I basically do it	6<br>I can do it	7<br>I can do it well
11	Can you leave a note for another student in English?					
1<br>I cannot do it at all	2<br>I cannot do it	3<br>Maybe I cannot do it	4<br>Maybe I can do it	5<br>I basically do it	6<br>I can do it	7<br>I can do it well
12	When you read English articles, can you guess the meaning of unknown words?					
1<br>I cannot do it at all	2<br>I cannot do it	3<br>Maybe I cannot do it	4<br>Maybe I can do it	5<br>I basically do it	6<br>I can do it	7<br>I can do it well
13	Can you make new sentences with the words just learned?					
1<br>I cannot do it at all	2<br>I cannot do it	3<br>Maybe I cannot do it	4<br>Maybe I can do it	5<br>I basically do it	6<br>I can do it	7<br>I can do it well
14	Can you write email messages in English?					
1<br>I cannot do it at all	2<br>I cannot do it	3<br>Maybe I cannot do it	4<br>Maybe I can do it	5<br>I basically do it	6<br>I can do it	7<br>I can do it well
15	If your English instructor gives you a tape-recorded English dialogue about everyday school matters, can you understand?					
1	2	3	4	5	6	7

I cannot do it at all		I cannot do it	Maybe I cannot do it	Maybe I can do it	I basically do it	I can do it	I can do it well
16	Can you understand the English news on the Internet?						
1 I cannot do it at all		2 I cannot do it	3 Maybe I cannot do it	4 Maybe I can do it	5 I basically do it	6 I can do it	7 I can do it well
17	Can you ask your English instructor questions in English?						
1 I cannot do it at all		2 I cannot do it	3 Maybe I cannot do it	4 Maybe I can do it	5 I basically do it	6 I can do it	7 I can do it well
18	Can you make sentences with English idiomatic phrases?						
1 I cannot do it at all		2 I cannot do it	3 Maybe I cannot do it	4 Maybe I can do it	5 I basically do it	6 I can do it	7 I can do it well
19	Can you introduce your English instructor to someone else in English?						
1 I cannot do it at all		2 I cannot do it	3 Maybe I cannot do it	4 Maybe I can do it	5 I basically do it	6 I can do it	7 I can do it well
20	Can you discuss subjects of general interest with your fellow students in English?						
1 I cannot do it at all		2 I cannot do it	3 Maybe I cannot do it	4 Maybe I can do it	5 I basically do it	6 I can do it	7 I can do it well
21	Can you read shot English narratives?						
1 I cannot do it at all		2 I cannot do it	3 Maybe I cannot do it	4 Maybe I can do it	5 I basically do it	6 I can do it	7 I can do it well
22	Can you understand English movies without subtitles?						
1 I cannot do it at all		2 I cannot do it	3 Maybe I cannot do it	4 Maybe I can do it	5 I basically do it	6 I can do it	7 I can do it well
23	Can you answer your English instructor's questions in English?						
1 I cannot do it at all		2 I cannot do it	3 Maybe I cannot do it	4 Maybe I can do it	5 I basically do it	6 I can do it	7 I can do it well
24	Can you understand English songs?						

1<br>I cannot do it at all	2<br>I cannot do it	3<br>Maybe I cannot do it	4<br>Maybe I can do it	5<br>I basically do it	6<br>I can do it	7<br>I can do it well
25	Can you read English newspapers?					
1<br>I cannot do it at all	2<br>I cannot do it	3<br>Maybe I cannot do it	4<br>Maybe I can do it	5<br>I basically do it	6<br>I can do it	7<br>I can do it well
26	Can you find out the meaning of new words by using English–English dictionaries?					
1<br>I cannot do it at all	2<br>I cannot do it	3<br>Maybe I cannot do it	4<br>Maybe I can do it	5<br>I basically do it	6<br>I can do it	7<br>I can do it well
27	Can you understand numbers spoken in English?					
1<br>I cannot do it at all	2<br>I cannot do it	3<br>Maybe I cannot do it	4<br>Maybe I can do it	5<br>I basically do it	6<br>I can do it	7<br>I can do it well
28	Can you write diary entries in English?					
1<br>I cannot do it at all	2<br>I cannot do it	3<br>Maybe I cannot do it	4<br>Maybe I can do it	5<br>I basically do it	6<br>I can do it	7<br>I can do it well
29	Can you understand English articles about Chinese culture?					
1<br>I cannot do it at all	2<br>I cannot do it	3<br>Maybe I cannot do it	4<br>Maybe I can do it	5<br>I basically do it	6<br>I can do it	7<br>I can do it well
30	Can you introduce yourself in English?					
1<br>I cannot do it at all	2<br>I cannot do it	3<br>Maybe I cannot do it	4<br>Maybe I can do it	5<br>I basically do it	6<br>I can do it	7<br>I can do it well
31	Can you write an essay in two pages about your English instructor in English?					
1<br>I cannot do it at all	2<br>I cannot do it	3<br>Maybe I cannot do it	4<br>Maybe I can do it	5<br>I basically do it	6<br>I can do it	7<br>I can do it well
32	Can you understand new reading materials (e.g., news from the Time magazine) selected by your English instructor?					
1<br>I cannot	2<br>I cannot	3<br>Maybe I	4<br>Maybe I	5<br>I basically	6<br>I can do it	7<br>I can do it

do it at all	do it	cannot do it	can do it	do it		well

S4. L2 WTC questionnaire

Please imagine how willing you would be to communicate in English in the following 10 situations. Think about your willingness to communicate in English and then choose the option that matches your feelings (1 = Definitely unwilling, 5 = Definitely willing). There are no right or wrong answers.				
1	When you are given an opportunity to talk freely in an English class.			
1 Definitely unwilling	2	3	4	5 Definitely willing
2	When you have an opportunity to talk in front of other students in an English class.			
1 Definitely unwilling	2	3	4	5 Definitely willing
3	When you have a group discussion in an English class.			
1 Definitely unwilling	2	3	4	5 Definitely willing
4	When you have an opportunity to make a presentation in front of a large group.			
1 Definitely unwilling	2	3	4	5 Definitely willing
5	When you have an opportunity to explain your own culture, in English, to your classmates.			
1 Definitely unwilling	2	3	4	5 Definitely willing
6	When you have an opportunity to talk with non-native speakers of English (e.g., German and Chinese) on social media.			
1 Definitely unwilling	2	3	4	5 Definitely willing
7	When you have an opportunity to talk with native speakers of English (e.g., American and British) on social media.			
1 Definitely unwilling	2	3	4	5 Definitely willing
8	When you have an opportunity to talk in English with other foreign fans in an online community (e.g., BTS, and Harry Potter).			
1	2	3	4	5

Definitely unwilling				Definitely willing
9	When you have an opportunity to talk in English with other game players.			
1 Definitely unwilling	2	3	4	5 Definitely willing
10	When you have an opportunity to explain your own culture online in English to other English speakers.			
1 Definitely unwilling	2	3	4	5 Definitely willing
